# Effect of Leukocyte-Rich and Platelet-Rich Plasma on Healing of a Horizontal Medial Meniscus Tear in a Rabbit Model

**DOI:** 10.1155/2015/179756

**Published:** 2015-06-09

**Authors:** Kyun Ho Shin, Haseok Lee, Seonghyun Kang, You-Jin Ko, Seung-Yup Lee, Jung-Ho Park, Ji-Hoon Bae

**Affiliations:** ^1^Department of Orthopaedic Surgery, Korea University Anam Hospital, Korea University College of Medicine & Medical School, Seoul 136-705, Republic of Korea; ^2^Korea University College of Medicine & Medical School, Seoul 136-705, Republic of Korea; ^3^Department of Orthopaedic Surgery, Korea University Ansan Hospital, Korea University College of Medicine & Medical School, Ansan 425-707, Republic of Korea; ^4^Department of Orthopaedic Surgery, Korea University Guro Hospital, Korea University College of Medicine & Medical School, Seoul 152-703, Republic of Korea

## Abstract

There are limited reports on the effect of platelet-rich plasma (PRP) on meniscus healing. The purpose of this study was to investigate the effect of leukocyte-rich PRP (L-PRP) on potential healing of the horizontal medial meniscus tears in a rabbit model. A horizontal medial meniscus tear was created in both knees of nine skeletally mature adult rabbits. Left or right knees were randomly assigned to a L-PRP group, or a control group. 0.5 mL of L-PRP from 10 mL of each rabbit's whole blood was prepared and injected into the horizontal tears in a L-PRP group. None was applied to the horizontal tears in a control group. The histological assessment of meniscus healing was performed at two, four, and six weeks after surgery. We found that there were no significant differences of quantitative histologic scoring between two groups at 2, 4, and 6 weeks after surgery (*p* > 0.05). This study failed to show the positive effect of single injection of L-PRP on enhancing healing of the horizontal medial meniscus tears in a rabbit model. Single injection of L-PRP into horizontal meniscus tears may not effectively enhance healing of horizontal medial meniscus tears.

## 1. Introduction

Platelet-rich plasma (PRP) is a concentrated source of a variety of growth factors that may enhance the healing of the bone and soft tissue. It can be simply obtained by centrifuging patient's whole blood, which makes the PRP easily applied to various musculoskeletal injuries. Although there is still insufficient evidence to support the use of PRP for treating these injuries [1], PRP has received much attention as a promising treatment for musculoskeletal injuries and the clinical use of PRP is increasing [2–4].

There are four families of PRP products based on their fibrin architecture and leukocyte content: pure platelet-rich plasma (P-PRP) family, leukocyte- and platelet-rich plasma (L-PRP) family, pure platelet-rich fibrin (P-PRF) family, and leukocyte- and platelet-rich fibrin (L-PRF) family [5, 6]. Among four families, the L-PRF is found to release growth factors slowly over 7 days [7, 8], suggesting that the combination of leukocytes and final architecture of the fibrin matrix considerably influence the growth factor trapping and release. In addition, they help growth factors to act as an assembly of platelets and leucocytes in a complex fibrin matrix [9–12]. Recently, L-PRF has promising results in trauma surgery of various fields including sports medicine and orthopedics experimentally and clinically [10, 13–15]. However, L-PRF cannot be used as injectable products due to their thick properties. Instead, L-PRP may be an alternative method in delivering PRP into the inside of the meniscus, because L-PRP is less thick than L-PRF.

Based on positive effects of PRP on cell proliferation, collagen synthesis, and vascularization, PRP has attempted to be used as a biologic augment for meniscus tissue regeneration [7, 16]. This approach should be promising, especially in young patients with symptomatic intrasubstance horizontal cleavage tears. Injection of PRP into the intrasubstance tear site may help healing of the torn meniscus and prevent the intrasubstance stable tears from further progression to unstable tears that require resection of meniscus. However, there are limited reports on the effect of L-PRP on the healing of a torn meniscus. The purpose of this study was to investigate the effect of leukocyte-rich PRP (L-PRP) on the healing of the horizontal medial meniscus tears in a rabbit model. We hypothesized that single injection of L-PRP into the tear site would enhance the healing of the horizontal medial meniscus tears compared to a L-PRP untreated group.

## 2. Materials and Methods

A local institutional animal care and use committee approved the present study.

### 2.1. Preparation of L-PRP

Nine 6-month-old skeletally mature adult male New Zealand white rabbits (3.0–3.5 kg) were used in the current study. Each rabbit was anesthetized using intramuscular injection of ketamine hydrochloride at a dose of 50 mg/kg body weight and 2% xylazine hydrochloride at a dose of 10 mg/kg body weight. After anesthesia, 10 mL whole blood samples from each rabbit were collected. L-PRP was prepared using the double-spinning approach with two centrifugation techniques [18]. 10 mL of whole blood was collected into tubes containing acid citrate dextrose. The sample tubes were then centrifuged using a centrifugation appliance (LZ-1730R, LABOGENE, Seoul, South Korea) at room temperature for 10 minutes at 2400 rpm to separate the blood components into three layers of RBCs, buffy coat, and platelet poor plasma (PPP). The PPP and buffy coat layers were carefully collected with a long cannula syringe and transferred to another tube. A second centrifugation was performed at room temperature for 15 minutes at 3600 rpm to concentrate the platelets. After second centrifugation, L-PPP layer was discarded and L-PRP was obtained. For each 10 mL of whole blood, the volume of L-PRP obtained was about 0.6–0.7 mL. The platelet and leukocyte concentration of L-PRP prepared from our method was checked from one of nine rabbits.

### 2.2. Surgical Procedures for a Meniscus Horizontal Tear Model

**Figure 1:**
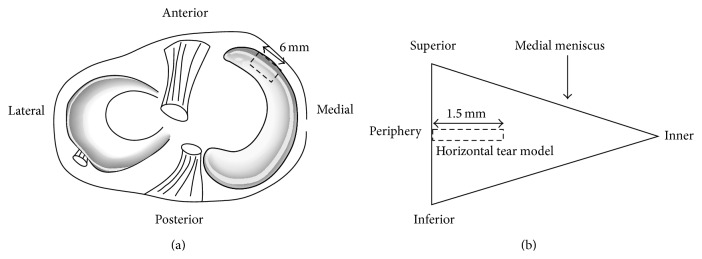
A horizontal tear in a rabbit model. A 6 mm in width and 1.5 mm in length of horizontal tear was created in the medial meniscus. (a) Tear location of the medial meniscus. (b) Radial plane of the medial meniscus.

After taking a sample of whole blood, both knees of each rabbit were operated by identical surgical techniques as a previous study [16]. All the procedures were performed through an aseptic technique. A medial parapatellar incision and arthrotomy were performed, the joint capsule was separated from synovial membranes, and the medial collateral ligament was exposed. Then the synovial membranes were cut transversely and released from the medial meniscus to expose the peripheral rim of the anterior horn of the meniscus. A horizontal tear model (6 mm in width and 1.5 mm in length) was created in the anterior horn of the medial meniscus with a number 11 surgical blade (Surgical Scalpel Blade number 11, Swann Morton Ltd., Sheffield, England) (Figure 1).

Left or right knees in each rabbit were randomly assigned to a L-PRP treated or a control group. To activate and release the growth factors, L-PRP was mixed with an equal volume of sterile saline solution containing 10% calcium chloride and 100 U/mL of sterile bovine thrombin before injection into the meniscus. Then, an aspiration needle (21 gauges) was directly inserted from the peripheral side of the created horizontal tear to the inner side of the meniscus. A form of L-PRP was injected into a horizontal tear area of the medial meniscus (L-PRP group). A total 0.5 mL of L-PRP was injected into the tear site with the injection rate of 50 *μ*L/sec in each rabbit. We performed this procedure before closing the knee joint because the created horizontal tear site was easily identified in the opened knee joint. None was applied to the tear in the opposite side of the knee (control group). The menisci were left unrepaired (stable tear). After injection, synovial tissue, joint capsule, and skin were sutured as separated layers in both knees in all rabbits. Intramuscular antibiotics were administered twice a day for 3 days in a row after surgery. All rabbits were returned to their cages and allowed to move freely during the experimental period without immobilization of both knees.

### 2.3. Histologic Evaluation

Rabbits were sacrificed at 2 weeks, 4 weeks, and 6 weeks postoperatively, and menisci were harvested from the knee joint and processed for histologic evaluation. The specimens were fixed in 4% paraformaldehyde in 0.1 M phosphate buffer solution for 4 h and embedded in paraffin wax. Each specimen was cut into 4 *μ*m thick slices along the radial plane (perpendicular to meniscus long axis). For histological analysis, sections were stained with hematoxylin and eosin staining and safranin-O fast green staining.

The length of a horizontal tear was measured using an image analysis software package (ImageJ, National Institute of Health, Maryland, USA).

Two independent observers evaluated healing of the meniscus tear using the two different quantitative scoring systems including the original quantitative scoring system [7] and semiquantitative scoring system which were used [16, 17]. The categories of original semiquantitative scoring system were modified in the present study. The assessment of “reparative tissues with bonding” was excluded because our study focused on the horizontal tear model of the meniscus rather than a circular defect model. The other two categories, “existence of fibrochondrocytes” and “stainability with safranin-O,” were evaluated. The total attainable score was 4 points from 2 for each category (Table 1). The other semiquantitative scoring system was based on whether the horizontal tear is regenerated or filled with fibrous tissue. The total attainable score of this scoring system was five points (Table 2). The points were calculated and analyzed statistically for each slide of specimen.

### 2.4. Statistical Analysis

SPSS version 12.0 (SPSS, IBM Corporation, Chicago, IL) was used to perform Wilcoxon signed rank-sum test for statistical analysis of all the results in the current study. Significance was set at *p* < 0.05.

## 3. Results

### 3.1. Concentration of Platelets in L-PRP

The concentration of platelets in L-PRP prepared from one of nine rabbits was checked as 2,482,000/*μ*L. This was at least 4 times more than previously reported normal value of platelets in whole blood of New Zealand white rabbits (338,000~602,000/*μ*L) [19] and it was more than the effective concentration of platelets in PRP (1,000,000/*μ*L) [20]. Also, the concentration of leukocytes in L-PRP was 140,400/*μ*L (neutrophil 62%, lymphocyte 32%, monocyte 5%) and is higher than previously reported normal range of leukocytes in whole blood of New Zealand white rabbits (4,200–12,300/*μ*L) [21].

### 3.2. Assessment of a Horizontal Tear Length

There was no statistically significant difference in the length of horizontal tear models between a control group (average 1.56 mm, range 1.26–1.61 mm) and a L-PRP group (average 1.51 mm, range 1.26–1.83 mm) (*p* > 0.05).

### 3.3. Histologic Assessment

**Figure 2:**
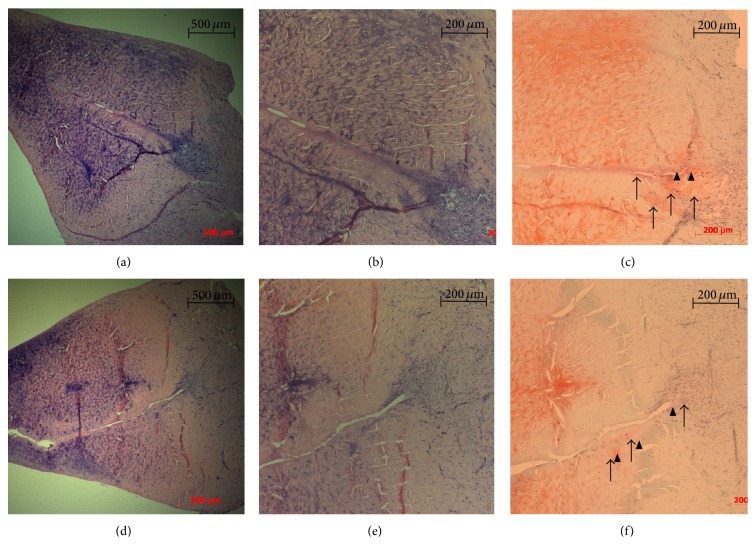
Histologic specimens of the medial meniscus tissues at 4 weeks after surgery. Sections (a), (b), and (c) are a control group. Sections (d), (e), and (f) are PRP group. Sections (a), (b), (d), and (e) are the sections stained with hematoxylin and eosin (H&E). Sections (c) and (f) are the sections stained with safranin-O. ((a) and (d)) In both groups, the meniscus tears were filled with fibrous tissue about 25% extending from peripheral area of the tears with localized existence of fibrochondrocytes. ((c) and (f)) The fibrous tissue was partially stained with safranin-O (black arrow) and chondrocyte-like cells were present (arrowhead) in peripheral area of the tears.

**Figure 3:**
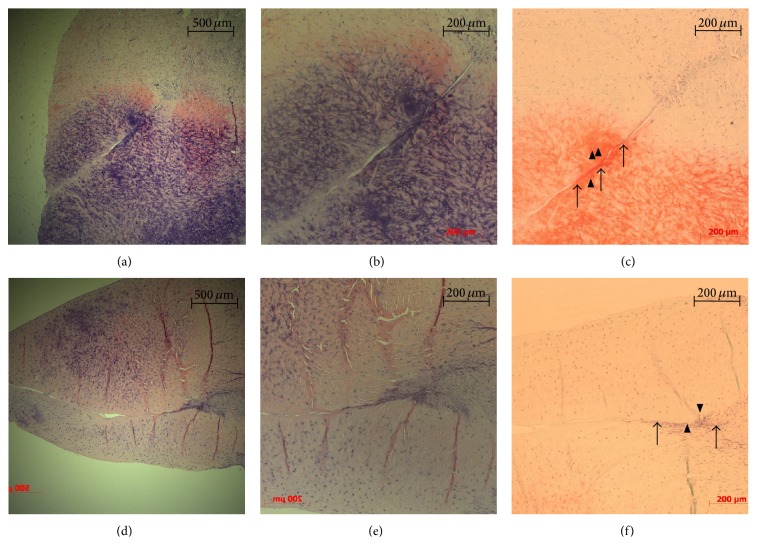
Histologic specimens of the medial meniscus tissues at 6 weeks after surgery. Sections (a), (b), and (c) are a control group. Sections (d), (e), and (f) are PRP group. Sections (a), (b), (d), and (e) are the sections stained with hematoxylin and eosin (H&E). Sections (c) and (f) are the sections stained with safranin-O. ((a) and (d)) In both groups, the meniscus tears were filled with fibrous tissue more than 50% extending from peripheral area of the tears with localized existence of fibrochondrocytes. ((c) and (f)) In both groups, the fibrous tissue was partially stained with safranin-O (black arrow) and some chondrocyte-like cells (arrowhead) were identified in peripheral area of the tears.

Reparative meniscus tissue, fibrochondrocytes, and dense staining of safranin-O were not prominent in a L-PRP group at 2, 4, and 6 weeks after surgery compared to a control group (Figures 2 and 3). Fibrochondrocytes were not plentiful but a few were found in both control and L-PRP groups 4 and 6 weeks after surgery. Also, safranin-O staining was not dense in both groups 4 and 6 weeks after surgery.

At 2 weeks after surgery, the meniscus tears were filled with fibrous tissue less than 25% extending from peripheral area of the tears with localized existence of fibrochondrocytes. The fibrous tissue was rarely stained with safranin-O and chondrocyte-like cells were not identified in a control group. The meniscus tears also showed safranin-O negative fibrous tissue less than 25% and no chondrocyte-like cells were identified in a L-PRP group.

At 4 weeks after surgery, a control group showed that the meniscus tears were filled with fibrous tissue about 25% extending from peripheral area of the tears with localized existence of fibrochondrocytes. The fibrous tissue was partially stained with safranin-O and some chondrocyte-like cells were identified in the peripheral area of the tears. In the L-PRP group, the meniscus tears showed about 25% of localized existence of fibrochondrocytes. The fibrous tissue was locally stained with safranin-O and some chondrocyte-like cells were identified (Figure 2).

At 6 weeks after surgery, in both control and L-PRP groups, the meniscus tears were filled with fibrous tissue more than 50% extending from the peripheral area of the tears with localized existence of fibrochondrocytes. The fibrous tissue was partially stained with safranin-O and some chondrocyte-like cells were identified in the peripheral area of the tears (Figure 3).

**Figure 4:**
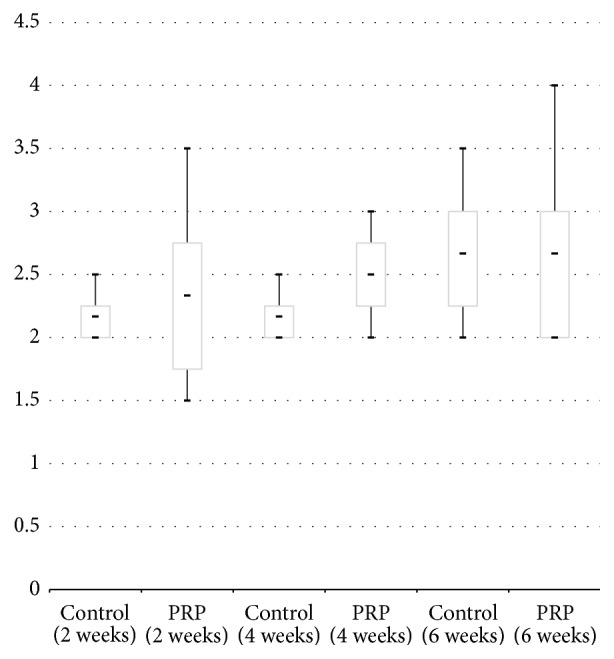
Box-plot diagram showing the results of modified original quantitative scoring at 2, 4, and 6 weeks after surgery. Control: untreated group, PRP: single injection of leukocyte-rich PRP treated group.

**Figure 5:**
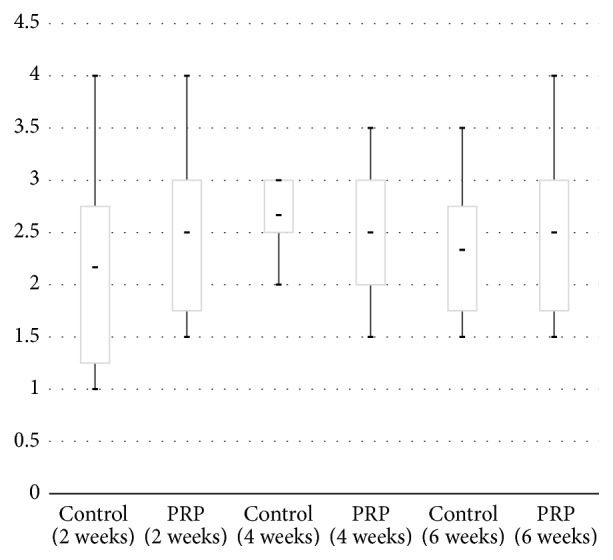
Box-plot diagram showing the results of semiquantitative scoring at 2, 4, and 6 weeks after surgery. Control: untreated group, PRP: single injection of leukocyte-rich PRP treated group.

The results of modified original quantitative scoring are shown in Figure 4. No significant differences of a mean score were observed between the control (2.2, 2.6, 2.3) and L-PRP group (2.5, 2.5, 2.5) at 2, 4, and 6 weeks after surgery (*p* value = 1.0, 0.655, and 1.0, resp.). The semiquantitative scoring system showed also no significant difference of a mean score between a control (2.2, 2.6, 2.3) and L-PRP group (2.3, 2.5, 2.6) at 2, 4, and 6 weeks after surgery (Figure 5, *p* value = 0.655, 0.655, 1.000, resp.).

## 4. Discussion

The present study investigated whether single injection of L-PRP into the torn meniscus could enhance the healing of the horizontal meniscus tears. We hypothesized that single injection of L-PRP would enhance the healing of the meniscus tears. However, quantitative histologic scoring results showed no significant differences of meniscus healing between a L-PRP treated group and a L-PRP untreated group. In contrast to our hypothesis, this study failed to show the positive effect of single injection of L-PRP on enhancing healing of the horizontal medial meniscus tears in a rabbit model.

There are previous in vivo studies reporting a positive effect of PRP or growth factors on the meniscus healing. Ishida et al. [22] investigated whether PRP enhances meniscal tissue regeneration in vitro and in vivo. They observed that PRP not only enhance proliferation of meniscal cells but also promoted in vitro the glycosaminoglycan synthesis. To test the in vivo effect, PRP with gelatin hydrogel (GH) was injected into the 1.5 mm diameter full thickness meniscus defect of the rabbits. They found that histologic scoring of the defect sites revealed significantly better meniscal repair in a PRP with GH treated group at 12 weeks after PRP injection, suggesting that PRP enhances the healing of meniscus defects. Furthermore, there was a tendency of higher scoring of a PRP group than a control group at 4 and 8 weeks, even though it was not statistically significant. Another study confirmed the positive effect of FGF-2, which is one of the growth factors released from platelets [16]. This study showed that GH incorporating FGF-2 enhanced the healing of meniscus horizontal tear (4 mm in width and 2 mm in length) in rabbits. At 4, 8, and 12 weeks after surgery, histologic healing scores were significantly higher in the GH with FGF-2 treated group than a GH without FGF-2 group.

One of the reasons for our different results from previous positive studies may be the difference of PRP delivery method. In order to promote the effect of growth factors in meniscus healing, the long exposure of growth factors to target area is important [12]. However, a half-life of growth factors is too short to maintain biologic activity in vivo. Therefore, the important issue is how to deal with the short half-life of growth factors when growth factors are locally applied to the meniscus tear area in vivo. Previously, Tabata et al. [23] proposed a controlled-release system using biodegradable acidic gelatin hydrogel as one of the methods to deal with the limitation of short half-life. In this system, growth factors are immobilized in gelatin hydrogels by physicochemical interaction with gelatin molecules, and the immobilized growth factors are released from the hydrogel as a result of hydrogel degradation. It was found that growth factors incorporated into the biodegradable acidic gelatin hydrogel can be slowly released and prevented from desorption; therefore, the target tissue can be continuously exposed to the growth factors for 2 weeks [9, 11]. Ishida et al. [22] observed that the hydrogel was left in the histologic slides after 4 weeks of the study, and they suggested that secretion of growth factor in PRP lasted at least 4 weeks. After 4 weeks of the surgery, fibrous tissues were abundant in the PRP groups compared to control group. Narita et al. [16] also used biodegradable gelatin hydrogel for PRP delivery in the meniscus horizontal tear. They found that FGF-2 with GH significantly stimulated proliferation of meniscal cells and inhibited the death of meniscal cells until 4 weeks, thereby increasing meniscal cell density and enhancing meniscus repair in a rabbit model. However, both studies [16, 22] did not measure directly the amount of growth factors released from the platelets in vivo; therefore, how long the growth factors maintained biologic activity is unclear.

The results of the current study showed no difference between the control and PRP group. The reason might be the earlier burst release and desorption of growth factors at meniscus tear. As we did not measure directly the amount of growth factors released from platelets, it is uncertain whether growth factors were appropriately released and activated or not. In addition, we are not unable to know how long growth factors maintained biologic activity. There is little information about in vivo release characteristics of growth factors because previous studies also did not measure the amounts of growth factors directly in the in vivo meniscus tear model [16, 22]. Further study will be required to measure the time-dependent amounts of various growth factors of L-PRP in the in vivo meniscus model. Another reason for negative results of this study may be possible inflammatory effect of leukocytes on the meniscus healing. One of advantages of the use of leukocyte-rich PRP is the slow and continuous release of growth factors (especially TGF-beta1) compared to pure PRP without leukocytes [6, 8]. However, recent studies have showed that the composition of L-PRP negatively affects the mechanical properties of the fibrin scaffolds and stimulates a more proinflammatory environment that is directly related to an increased cell-inflammatory condition and a reduced cell proliferation response, which ultimately may be detrimental for tissue regeneration [24–26]. Even though our study showed no inflammatory reaction in all specimens, negative effect of leukocytes on meniscus healing can be considered for our results.

Ehrenfest et al. [7] showed that safranin-O stained tissues were not found in all specimens until 12 weeks after surgery. In our study, safranin-O stained tissues were found in both groups at 4 weeks after surgery and it was also observed in the control group at 6 weeks after surgery. One possible explanation is an existence of the vascular access channels. Arnoczky and Warren [27] reported that when the vascular access channels connecting the peripheral vascular zone with the inner avascular zone are made, the injury site in the avascular zone can be exposed to the vascular originated growth factors and promote meniscal regeneration. The horizontal tear models in both control and PRP groups are likely to have the vascular access channels that connect the peripheral vascular area with inner avascular area of the meniscus. Therefore, not only a PRP group but also a control group might be exposed to a certain level of growth factors. It is assumed that fibrochondrocytes have migrated towards chemotactic growth factors that were released from either peripheral blood vessels or PRP.

Our results should be interpreted with the following limitations. First, as mentioned above, as we did not measure directly the amount of growth factors released from platelets, it is uncertain whether growth factors were appropriately released and activated or not. In addition, we are not unable to know how long growth factors maintained biologic activity. Therefore, we could not explain the reasons for negative results exactly. Second, meniscus specimens were sectioned perpendicular to their longitudinal axis for histologic evaluation. With these slides, meniscus healing could be evaluated in a radial direction including meniscocapsular junction to inner avascular area. However, we did not evaluate the meniscus healing in a longitudinal plane. Third, the evaluation time points were 2, 4, and 6 weeks after surgery. Therefore, the effect of L-PRP injection on the meniscus healing over 6 weeks after surgery is not known. Fourth, unlike Ishida et al.'s study that implanted PRP incorporated with hydrogel sheet to the circular defect model [22], L-PRP was injected from peripheral to inner avascular zone through narrow tears. Therefore, it is uncertain that PRP was evenly distributed and captured within the meniscus. In the future study, it will be needed to develop a method to sufficiently deliver PRP to the target area through narrow meniscal tears. Finally, a sample size was too small to detect differences of histologic scoring system between two groups. Power analysis should be performed for appropriate sample size. However, we believe that descriptive results of histologic findings help us assess the effect of L-PRP on the meniscus healing.

## 5. Conclusions

This study failed to show the positive effect of single injection of L-PRP on enhancing healing of the horizontal medial meniscus tears in a rabbit model. Single injection of L-PRP into horizontal meniscal tears may not effectively enhance healing of the horizontal medial meniscus tears.

## Figures and Tables

**Table 1:** Modified original quantitative scoring system [7].

Category	Points
(1) *Existence of fibrochondrocytes *	
Fibrochondrocytes exist diffusely in the reparative tissues	2
Fibrochondrocytes are localized in the reparative tissues	1
Fibrochondrocytes are not found in the reparative tissues	0
(2) *Staining with safranin-O *	
Densely stained with safranin-O	2
Faintly stained with safranin-O	1
Not stained with safranin-O	0

**Table 2:** Semiquantitative scoring system [16, 17].

Category	Points
Complete regeneration without any evidences of meniscal tear	5
Full regeneration with location of the tear identified	4
Entire infiltration with fibrous tissue	3
Infiltration with fibrous tissue more than 25% of the tear	2
Infiltration with fibrous tissue 25% or less of the tear	1
No evidence of fibroblastic proliferation	0
